# Human retinal organoid model of disease-relevant photoreceptor cell death amenable to drug screening

**DOI:** 10.1038/s41419-026-08724-y

**Published:** 2026-04-13

**Authors:** Shama Parween, Anthony J. Saviola, Anna C. Howell, Stefanie Varghese, David Ceja Galindo, M. Natalia Vergara

**Affiliations:** 1 CellSight Ocular Stem Cell and Regeneration Research Program, Sue Anschutz Rodgers Eye Center, University of Colorado School of Medicine, Aurora, CO USA; 2 Department of Biochemistry and Molecular Genetics, University of Colorado School of Medicine, Aurora, CO USA; 3 Linda Crnic Institute for Down Syndrome, University of Colorado Anschutz Medical Campus, Aurora, CO USA; 4 University of Colorado Alzheimer’s and Cognition Center, University of Colorado School of Medicine, Aurora, CO USA

**Keywords:** Stem cells, Disease model

## Abstract

Dry age-related macular degeneration (AMD) is characterized by the progressive loss of retinal pigment epithelium cells in the macula, leading to photoreceptor degeneration and loss of central vision. Current treatments only modestly delay disease progression, but once photoreceptors are damaged, vision loss becomes irreversible. Therefore, there is an urgent need to develop therapies that prevent photoreceptor cell death and that may complement current and emerging treatment strategies. A critical step toward this goal is establishing pathophysiologically relevant human disease models for therapeutic testing. In this study, we developed a human induced pluripotent stem cell-derived retinal organoid (RO) model that recapitulates key aspects of AMD-associated photoreceptor degeneration. To mimic environmental stressors relevant to AMD, we treated mature ROs with cigarette smoke extract (CSE), a known oxidative agent and major modifiable risk factor for the disease. CSE exposure induced oxidative stress, mitochondrial membrane depolarization, and cell death primarily in the outer nuclear layer. Photoreceptor degeneration in this model involves the activation of the intrinsic apoptotic pathway and ferroptosis, which is accompanied by lipid peroxidation and dysregulation of the glutathione system. Proteomic profiling confirmed alterations in metabolic, redox, and cell death pathways consistent with AMD pathophysiology, and offered further insight into the mechanistic interplay among these pathways. Furthermore, we integrated this model with robust, quantitative outcome measures in live ROs, offering a powerful platform for preclinical therapeutic screening in dry AMD.

## Introduction

Age-related macular degeneration (AMD) is one of the leading causes of vision loss and blindness in people over the age of 50 worldwide [1, 2]. In AMD, the retinal pigment epithelium (RPE) in the macular region of the retina degenerates, followed by progressive photoreceptor death and loss of central vision [3, 4]. The disease is categorized into neovascular or “wet” AMD and non-neovascular or “dry” AMD, the latter comprising approximately 90% of AMD cases.

The etiology of AMD is complex, encompassing a combination of modifiable and non-modifiable risk factors. Non-modifiable risk factors include Caucasian ethnicity and certain genetic polymorphisms, particularly in genes involved in the complement pathway, but the major non-modifiable risk factor associated with AMD is age [5–7]. Conversely, smoking is the most significant modifiable environmental risk factor for AMD, accelerating the onset and increasing the progression of the disease by two- to fivefold [7–9]. Cigarette smoke contains potent oxidants and free radicals that are absorbed in the bloodstream through the lungs and reach the retina, a tissue with high metabolic demand. This leads to oxidative damage, particularly to lipids and mitochondria, contributing to retinal degeneration [2]. Since mitochondria are crucial for ATP production and cellular metabolism, their damage disrupts cellular homeostasis, contributing to RPE degeneration, photoreceptor dysfunction, and apoptotic death [10–14]. Iron released from cigarette smoke can also exacerbate lipid peroxidation, leading to cell membrane damage and ferroptosis, a form of regulated cell death that has been implicated in the pathogenesis of AMD [14–16]. Additional environmental risk factors include UV light exposure, which causes photooxidative damage, chronic inflammation, and high-fat diets [17].

The combination of these insults affects both the RPE and the retina. When the RPE is damaged, it cannot provide adequate support to the photoreceptors, leading to their dysfunction and death. Additionally, photoreceptor cells can display atrophic changes that precede observable changes in the RPE and choroid, sometimes by decades, and photoreceptor segment thinning has been recently proposed as an early predictor of AMD risk [18, 19]. Ultimately, once photoreceptors degenerate, vision loss becomes irreversible.

There are currently no cures for dry AMD, and the few treatments available to slow down RPE lesion expansion display limited efficacy in preserving vision [20, 21]. Therefore, there’s a critical need for treatment strategies that can preserve retinal photoreceptors and complement RPE-targeted treatments. Moreover, the success of RPE transplantation strategies currently in clinical trials depends on the presence of viable photoreceptor cells.

Multiple in vitro and in vivo models that mimic RPE dysfunction in AMD have been used for decades to investigate disease pathophysiology and evaluate therapeutic strategies [22, 23]. However, the field is lagging in the development of adequate research models to evaluate the impact of AMD-relevant insults on photoreceptors, which are required for the evaluation of photoreceptor preservation strategies. Particularly, there’s a need for validated human-based models that can recapitulate aspects of human pathophysiology and complement findings from animal studies.

In this context, retinal organoids (RO) derived from human induced pluripotent stem cells (hiPSC) open new opportunities. ROs recapitulate the three-dimensional structure and cellular composition of the native retina, including the presence of photoreceptors with inner and outer segments and light responsivity [24]. Therefore, we set out to develop and characterize hiPSC-derived retinal organoid models of photoreceptor degeneration that mimic critical aspects of AMD pathophysiology, and to validate assays for their application to drug screening. To accomplish this, we devised a strategy based on the treatment of human ROs with cigarette smoke extract (CSE) and assessed its impact on oxidative stress, mitochondrial health, cell death mechanisms, and overall impact on the retinal proteome. We optimized the concentration and timing of CSE treatment to preferentially target photoreceptor cells, and developed fast and quantitative fluorescence-based assays for live ROs that can be applied to the evaluation of drug efficacy and retinal toxicity. Finally, since the CSE product used in this research is a standardized but chemically complex mixture, we also generated initial data on the feasibility of H_2_O_2-_ or NaIO_3_-induced retinal degeneration as two chemically-defined alternative paradigms.

These human-based models and technologies provide new tools for the development of photoreceptor-targeted therapeutics in a human context, which can accelerate the development of much-needed therapies for dry AMD.

## Results

### Induction of cell death in human retinal organoids

We generated ROs from three different hiPSC lines using a well-characterized protocol [24, 25], and differentiated them in culture for 180 days (Fig. S1A). Immunofluorescence staining demonstrated the presence of major retinal cell types, as previously described [24, 25] (Fig. S1B). We chose 180 days of differentiation (D180) as a suitable time point for follow-up experiments because ROs of this age contain relatively mature rod and cone photoreceptors with inner and outer segment structures and light responsivity, allowing us to approximate the adult human scenario [24, 26–30]. Moreover, RNA-sequencing studies by multiple groups have found that late-stage human retinal organoids cluster with adult human retinal tissue, highlighting the relevance of ROs at this differentiation timepoint to model diseases of the adult retina [31–33].

**Fig. 1:**
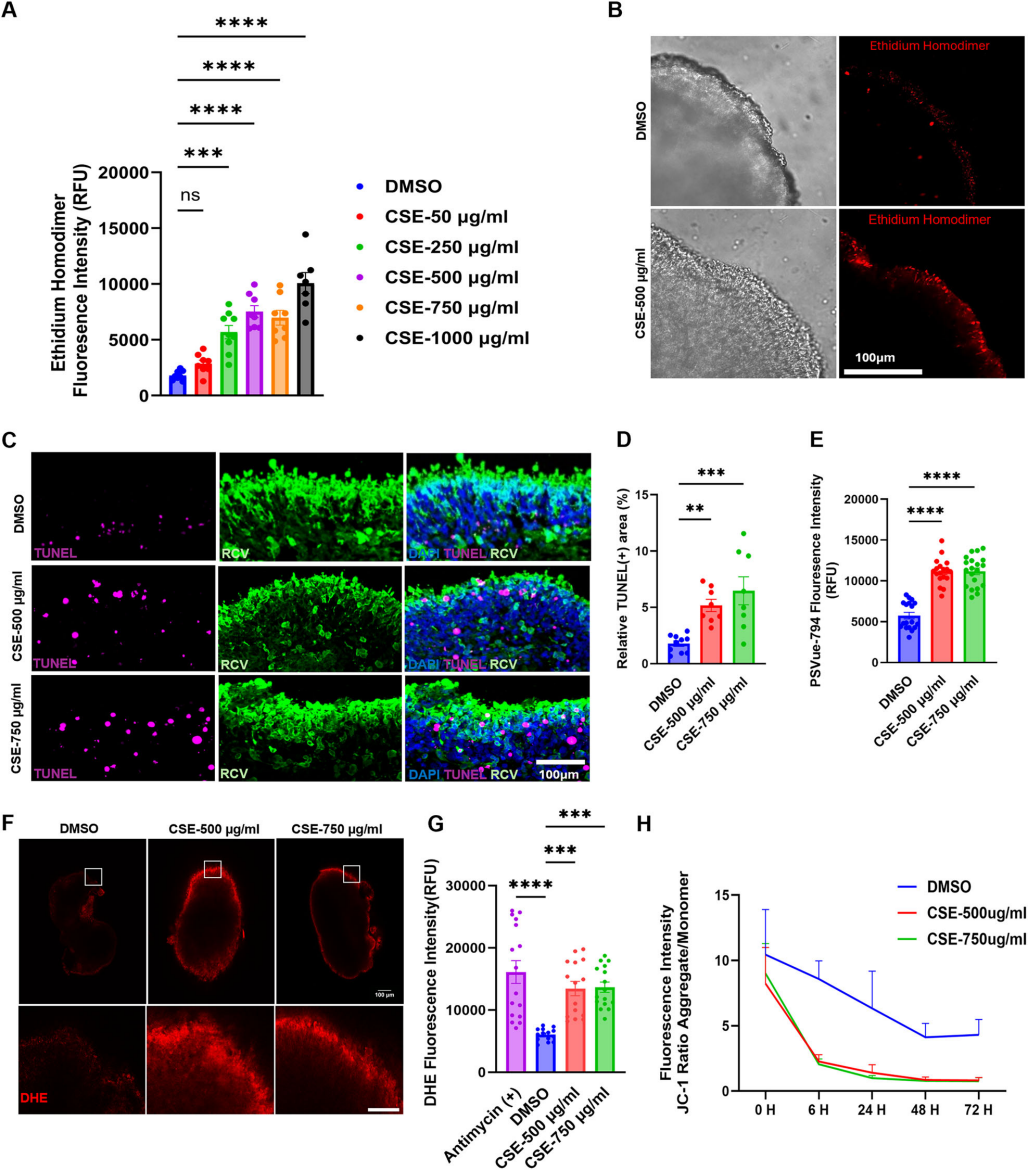
Effect of CSE on retinal cell death and cellular damage. **A** Retinal organoids were treated with increasing concentrations of CSE (50–1000 µg/ml) for 48 h, and cell death was assessed in live organoids using ethidium homodimer. Error bars represent Mean ± SEM, One-way ANOVA; ^****^p < 0.0001; ^***^p < 0.002; ns= non-significant. *N* = 8-10 ROs/condition. **B** Photomicrographs illustrate bright field and fluorescence images of whole-mount, live ROs incubated with ethidium homodimer. Scale bars represent 100 µm. **C** Representative confocal micrographs of sections of D180 RO treated for 48 h with CSE 500 or 750 µg/ml or vehicle (DMSO) control and stained with TUNEL to examine apoptosis-induced DNA fragmentation, along with recoverin (REC) staining to identify photoreceptor cells. Scale Bar: 100 µm. **D** TUNEL staining quantification shows a statistically significant increase in CSE-treated ROs compared to DMSO. Relative TUNEL (+) area: DMSO: 1.78 ± 0.24 (*n* = 10), CSE-500 µg/ml: 5.17 ± 0.54 (*n* = 8), and CSE-750 µg/ml: 6.47 ± 1.24 (*n* = 8). Error bars indicate Mean ± SEM; ^**^p < 0.005; ^***^p < 0.0003, One way ANOVA. **E** Quantification of fluorescence intensity (RFU) of PSVue-794 in live ROs under each condition to assess PS externalization as an early indicator of apoptosis. CSE induces a statistically significant increase in PSVue794 intensity compared with vehicle (DMSO) control. DMSO = 5740.92 ± 394.91 (*n* = 18), CSE-500µg/ml= 11147.67 ± 374.55, and CSE-750µg/ml=11175.55 ± 412.97 (*n* = 20) Mean ± SEM; ^****^p < 0.0001, One way ANOVA. **F** Confocal images of live retinal organoids at D180 showing increased dihydroethidium (DHE) positive cells in CSE-treated ROs compared to vehicle (DMSO) controls. Scale bar: 100 µm. **G** Quantification of DHE fluorescent intensity (RFU) in live ROs at D180 under each condition, including the Antimycin (+) control. Bar graph shows a statistically significant increase in ROS in CSE-treated ROs compared to vehicle (DMSO) controls. Relative RFU: Antimycin= 16123.75 ± 1841.83 (*n* = 16), DMSO: 6046.92 ± 266.07 (n = 14), CSE-500 µg/ml=13458.66 ± 1144.74 (*n* = 15), and CSE-750µg/ml=1366.5 ± 830.142 (*n* = 16). Error bars indicate Mean ± SEM; ^***^p < 0.0002, One way ANOVA. **H** Mitochondrial membrane depolarization in live ROs at D180 was assessed by JC-1 staining. Fluorescence quantification of JC-1 aggregate/monomer ratios was assessed longitudinally at 0, 6, 24, 48 and 72 h. in organoids treated with vehicle (DMSO) control or CSE (500 µg/ml and 750 µg/ml. Error bars indicate Mean ± SEM. *N* = 24–27 independent biological replicates per condition. ^****^p < 0.0001, Two-way ANOVA.

CSE has been extensively used to model AMD-like conditions in RPE cultures, and smoking chambers are used in in-vivo mouse models of AMD to study disease pathophysiology [34–36]. Therefore, we decided to test CSE exposure as a paradigm of photoreceptor degeneration in ROs. We conducted a CSE dose-response experiment for 48 h, and evaluated the outcomes in live ROs via ethidium homodimer staining, a commonly used cell death assay that fluorescently labels the nuclei of cells that have lost plasma membrane integrity (Fig. 1A, B). To quantify the results, we used a technique we previously developed termed 3D-automated reporter quantification (3D-ARQ), which allows for fast and quantitative analysis of fluorescence intensity in 3D organoids [37]. This technique uses a microplate reader with Z-dimensional focus and precise wavelength selection capabilities, and was shown to meet the speed, sensitivity and quantitative capacity required for high-throughput applications [37]. The results showed a concentration-dependent effect on retinal organoid viability and integrity. Lower concentrations (≤250 µg/ml) caused minimal cellular stress, while higher concentrations (1000 µg/ml) led to acute toxicity (Fig. 1A), accompanied by extensive structural disruption and outer segment deterioration (not shown). Based on these results, we selected intermediate concentrations of CSE (500 µg/ml and 750 µg/ml) for subsequent studies, as they elicited measurable cellular stress and cell death without complete loss of organoid architecture.

To validate these findings, we performed TUNEL staining on RO cryosections, which revealed a statistically significant increase in DNA fragmentation, a hallmark of late-stage apoptosis, in ROs treated with CSE at both concentrations for 48 h, compared to controls (Fig. 1C, D). Extended treatment with 500 µg/ml CSE for 5 days further increased the extent of cell death (S.2A, B). Cell death was particularly pronounced in the outer nuclear layer, identified by recoverin (RCV) photoreceptor immunostaining (Figs. 1C and S2A).

Additionally, we evaluated phosphatidyl serine (PS) externalization in live ROs using a PSVue-794 probe and 3D-ARQ [38, 39]. PSVue-794 is a positively charged bis (zinc-dipicolylamine) fluorescent probe that binds to exposed anionic phospholipids such as PS, which is externalized during the early stages of apoptosis or necrosis. We found that CSE treatment significantly increased PSVue-794 signal in CSE-treated ROs at both 500 µg/ml and 750 µg/ml concentrations for 48 h compared to vehicle-treated controls (Fig. 1E). Ultimately, we chose to continue our characterization of the model with the 48-h treatment length, due to the increased photoreceptor toxicity associated with longer durations of CSE treatment.

Finally, considering the chemical complexity of CSE, we decided to compare its effects with those of other known oxidative stressors: hydrogen peroxide (H₂O₂, 1 mM) and sodium iodate (NaIO₃, 0.5 mM) [40, 41]. We found that both of these stressors also induced a significant increase in cell death assessed by either PsVue-794 or ethidium homodimer and quantified by 3D-ARQ (Fig. S3A–D). This not only validates the findings of our CSE model but also provides two alternative, chemically-defined paradigms of retinal damage in human organoids that can be used for the evaluation of neuroprotective strategies.

### CSE treatment induces oxidative damage and mitochondrial dysfunction in human ROs

Oxidative stress and mitochondrial dysfunction have been strongly associated with AMD pathogenesis [42–44]. Recent studies suggest that photoreceptors and RPE have an interconnected metabolism and that mitochondrial damage triggers the degeneration of both RPE and photoreceptors in AMD [45]. In order to assess whether CSE induces oxidative stress in our model, we measured reactive oxygen species (ROS) levels in live ROs using a DHE assay and 3D-ARQ [41]. DHE is a cell-permeable red-fluorescent dye used for the detection of superoxide and hydrogen peroxide as indicators of oxidative stress in live cells [46]. We found that the CSE-treated ROs exhibit a significant increase in DHE fluorescence primarily in the outer nuclear layer, compared to vehicle-treated controls (Fig. 1F). Antimycin-A, an inhibitor of complex III of the mitochondrial electron transport chain, was used as a positive control. Our results indicate a significant increase in ROS production in CSE-treated ROs at both concentrations compared to vehicle-treated controls (Fig. 1G). Further, the oxidative effects of CSE were comparable to those induced by H₂O₂ and NaIO₃, in which DHE quantification indicated increased ROS production compared to control conditions (Fig. S3E, F).

Considering that mitochondrial health is crucial in protecting against oxidative stress-induced degradation [41], we evaluated whether CSE treatment induces mitochondrial stress or damage in our model using the JC-1 assay. JC-1 is a dye that demonstrates a potential-dependent accumulation in mitochondria. At lower membrane potentials, the dye presents in its monomeric state, emitting green fluorescence at ~529 nm. When the membrane potential is high, the dye aggregates, shifting to red fluorescence wavelengths at ~590 nm. Consequently, a decreased ratio of red to green fluorescence indicates mitochondrial depolarization. Notably, this assay has previously been used to selectively evaluate mitochondrial health in photoreceptor cells in retinal explants, primary retinal cultures, and retinal organoids [37, 47]. Thus, we performed a longitudinal quantitative analysis in live ROs treated with 500 or 750 µg/ml CSE or vehicle control. Our results revealed a significant decrease in the JC-1 aggregate/monomer fluorescence ratio in CSE-treated ROs after 6, 24, 48, and 72 h of treatment compared to controls (Fig. 1H), indicating that CSE induces mitochondrial dysfunction in human ROs consistent with AMD pathophysiology. Overall, these findings confirm that CSE triggers oxidative damage and cell death pathways in retinal organoids, underscoring its relevance as a model for environmentally induced retinal stress.

### CSE-driven photoreceptor cell death in ROs involves intrinsic apoptotic pathway activation

**Fig. 2:**
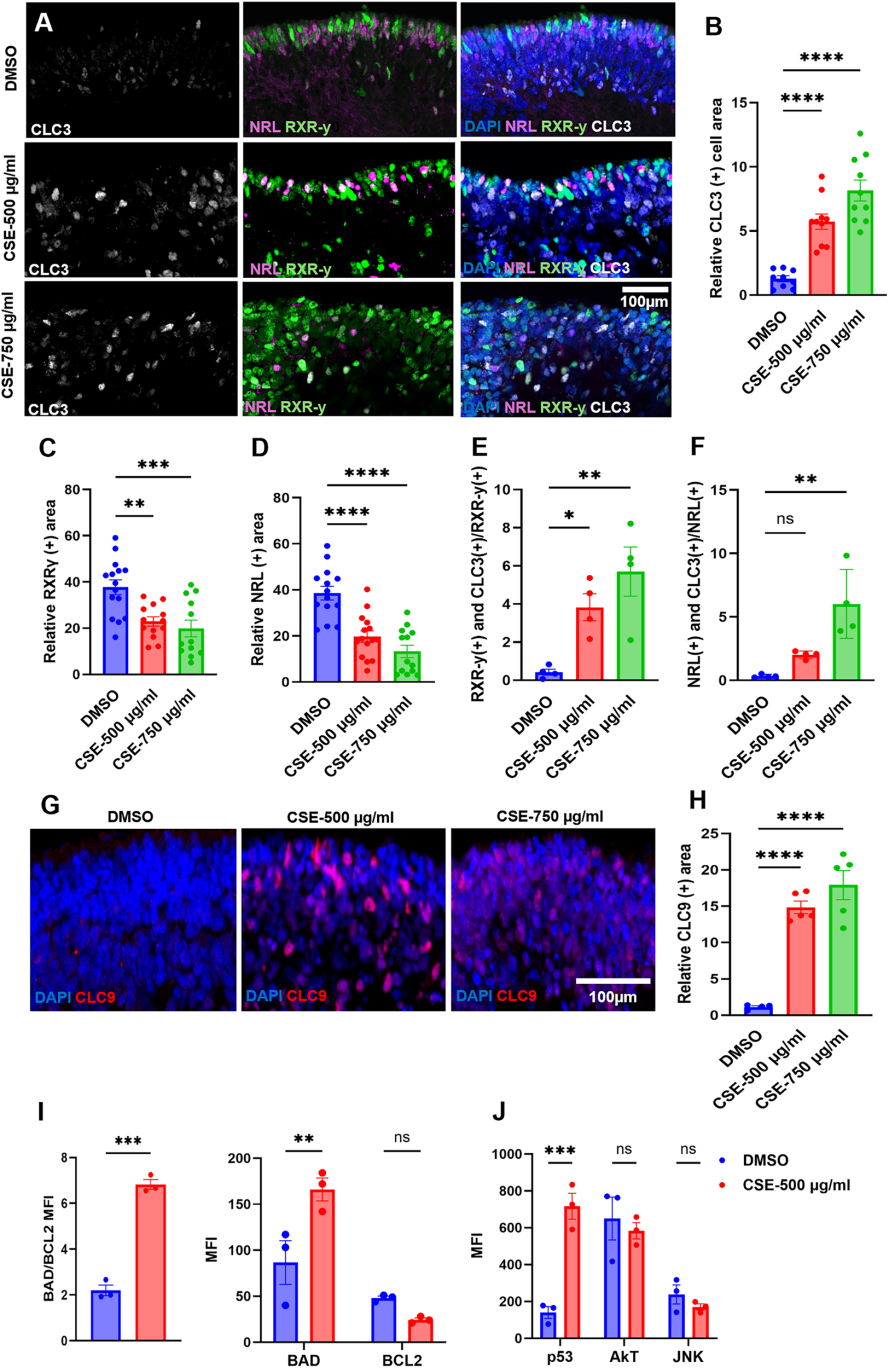
Evaluation of apoptotic mechanisms induced by CSE in retinal organoids. **A** Immunostaining of sections from D180 ROs treated with CSE at 500 or 750 µg/ml or vehicle (DMSO) control, using antibodies for cleaved caspase-3 (CLC3) and photoreceptor markers NRL (rod) and RXR-γ (cone). Scale bar: 100 µm. **B** Quantification of the relative cleaved caspase-3-labeled area indicates a statistically significant increase in CLC3-positive cells in CSE-treated ROs. DMSO = 1.265 ± 0.22 (*n* = 9), CSE-500 µg/ml= 5.723 ± 0.59 (*n* = 10) and CSE-750 µg/ml=8.152 ± 0.82 (*n* = 10). Error bar represents Mean ± SEM; One way ANOVA; ^****^p < 0.0001. **C** Quantification of RXR-γ (+) cells/DAPI indicates a statistically significant decrease in number of cone photoreceptors in CSE-treated ROs compared to vehicle (DMSO) controls. DMSO = 37.740 ± 3.17 (*n* = 15), CSE-500 µg/ml=22.874 ± 1.99 (*n* = 13), and CSE-750 µg/ml=19.957 ± 3.62 (*n* = 12). Error bars represent Mean ± SEM. One way ANOVA; ^**^p < 0.002; ^***^p < 0.002; ^*^p < 0.0005. **D** Quantification of NRL (+) cells/DAPI indicates a statistically significant decrease in number of rod photoreceptors in CSE-treated ROs compared to vehicle (DMSO) controls. DMSO = 38.590 ± 2.98 (*n* = 14), CSE-500 µg/ml=19.712 ± 2.45 (*n* = 15), and CSE-750 µg/ml=13.282 ± 2.71 (n = 13). Error bars represent Mean ± SEM. One way ANOVA; ^****^p < 0.0001. **E** Bar graph represents the percentage of RXR-γ (+)-ve cells in the outer nuclear layer that co-localize with ClC3 immunostaining. DMSO = 0.418 ± 0.15 (*n* = 4), CSE-500µg/ml=3.825 ± 0.70 (*n* = 4) and CSE-750 µg/ml=5.702 ± 1.28 (*n* = 4). Error bar shows Mean ± SEM. One way ANOVA; ^*^p < 0.05; ^**^p < 0.005. **F** Bar graph represents the percentage of NRL (+)-ve cells that co-localize with ClC3 immunostaining. DMSO = 0.334 ± 0.06 (*n* = 4), CSE-500µg/ml=2.007 ± 0.15 (*n* = 4) and CSE-750 µg/ml=6.023 ± 1.35 (*n* = 4). Error bars represent Mean ± SEM. One way ANOVA; ns=non-significant; ^**^p < 0.002. **G** Immunostaining on RO sections shows an increase in cleaved caspase-9 staining in CSE-treated ROs compared to (DMSO) vehicle controls, indicating activation of the intrinsic apoptotic pathway. Scale bar: 100 µm. **H** Quantification of relative cleaved caspase-9-labeled area/DAPI shows a significant increase in CLC9-stained cells in CSE-treated ROs compared to vehicle (DMSO) controls. DMSO = 1.136 ± 0.17 (*n* = 4), CSE-500µg/ml=14.839 ± 0.85 (*n* = 5) and CSE-750 µg/ml=17.898 ± 1.98 (*n* = 5). Error bars show Mean ± SEM. One-way ANOVA; ^****^p < 0.0001. **I** Multiplex ELISA on protein extracts from ROs treated with CSE 500 µg/ml or vehicle (DMSO) control shows a statistically significant increase in pro-apoptotic BAD and in the BAD/BCL2 ratio induced by CSE. BAD: DMSO = 86.667 ± 23.68 (*n* = 3) and CSE-500 µg/ml=166.00 ± 12.49; BCL2; DMSO = 48.00 ± 2.08 (*n* = 3),and CSE-500 µg/ml=24.333 ± 2.02 (*n* = 3); BAD/BCL2 ratio: DMSO = 2.19 ± 0.23 (*n* = 3),and CSE-500 µg/ml=6.822 ± 0.21 (*n* = 3). Error bars represent Mean ± SEM. *T*-test (Unpaired); and One-way ANOVA; ^**^p < 0.005; ^***^p < 0.0001. **J** CSE treatment increased the levels of p53, which activates the DNA damage response pathway. p53: DMSO = 140.000 ± 31.72 (*n* = 3),and CSE-500 µg/ml=716.667 ± 70.59 (n = 3); AkT: DMSO = 650.000 ± 0116.51 (*n* = 3),and CSE-500 µg/ml=583.333 ± 43.899 (*n* = 3); and JNK DMSO = 238.000 ± 51.59 (n = 3), and CSE-500 µg/ml=170.333 ± 17.13 (*n* = 3). Error bars represent Mean ± SEM. *T*-test (Unpaired); and One-way ANOVA; ^**^p < 0.005; ^***^p < 0.0001.

Numerous studies have highlighted the role of apoptosis as a major driver of photoreceptor cell death in AMD [12, 48, 49]. To investigate the involvement of apoptotic mechanisms in our model, we performed immunostaining on cryosections of d180 ROs treated for 48 h with CSE or vehicle, using a cleaved caspase3 (CLC3) antibody combined with NRL (rod) and RXR-γ (cone) as photoreceptor markers. We observed a significant increase in CLC3 staining in CSE-treated ROs compared to controls (Fig. 2A, B). Notably, the majority of CLC3-positive cells colocalized with NRL and RXR-γ (Fig. 2E, F), which led to an overall decrease in the proportions of rods and cones in CSE-treated retinas (Fig. 2C, D). We also observed a significant increase in cleaved caspase-9 (CLC9) activation in CSE-treated ROs, suggesting the involvement of the intrinsic apoptotic pathway (Fig. 2G, H). This was further supported by our MLLIPLEX assay, which showed significantly increased pro-apoptotic Bad protein levels, a trend towards decreased pro-survival Bcl2 protein levels, and a significant increase in the Bad/Bcl-2 ratio in CSE-treated ROs compared to controls (Fig. 2I). We also observed an upregulation of the tumor suppressor protein p53 (Fig. 2J), which plays a role in the induction of apoptosis through the intrinsic pathway and has been shown to participate in rod photoreceptor degeneration in mouse models and the in age-dependent degeneration of human RPE cells [50, 51]. Conversely, we did not observe differences in pAkt or JNK levels between CSE-treated ROs and vehicle-treated controls.

Overall, these results support the hypothesis that CSE-mediated photoreceptor cell death in our model involves the recruitment of the intrinsic apoptotic pathway, which correlates with the mitochondrial damage and oxidative stress we observed upon CSE treatment.

### CSE treatment induces ferroptosis in human ROs

**Fig. 3:**
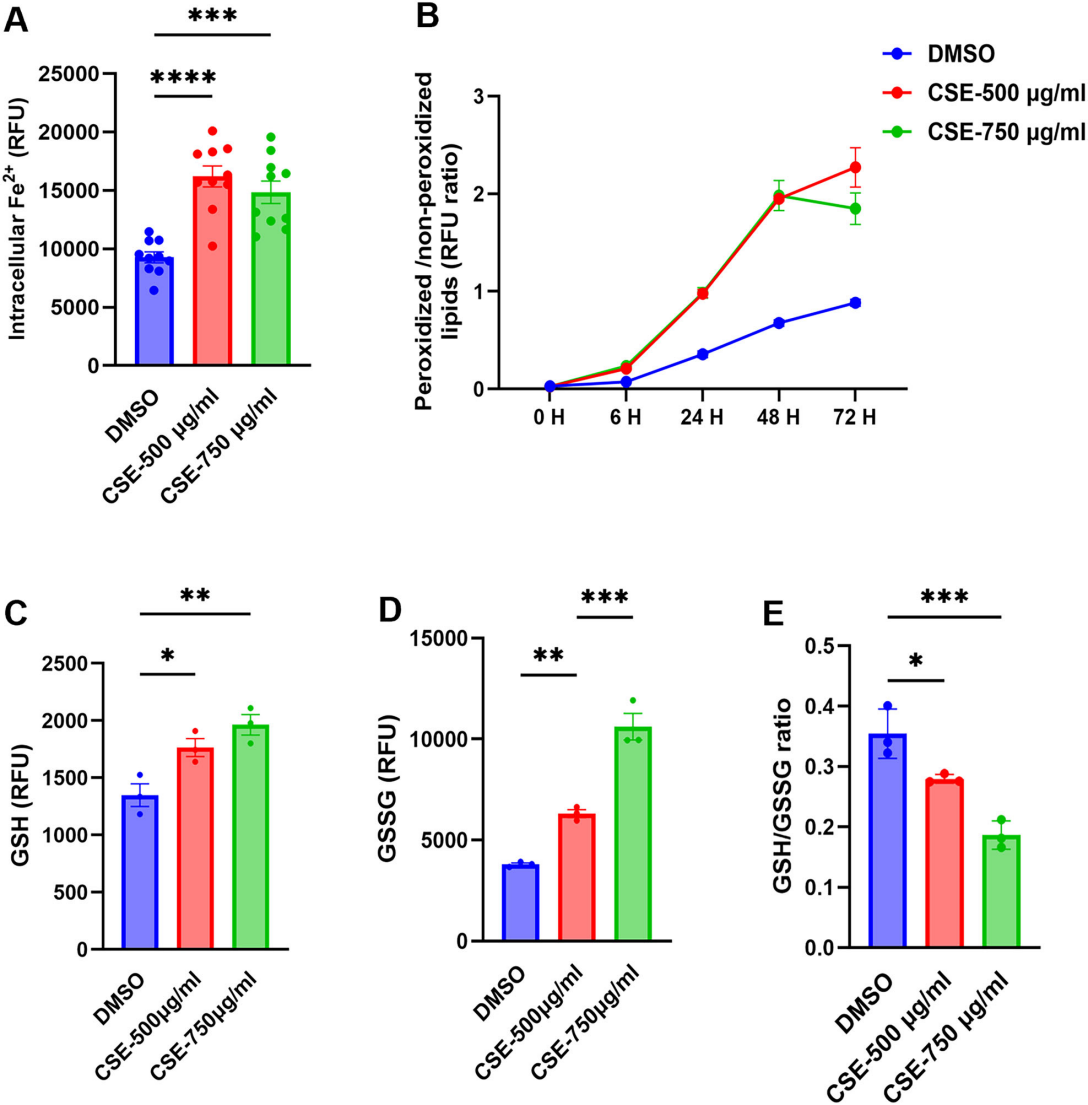
CSE treatment induces ferroptosis in human ROs. **A** Live, D180 ROs were treated for 48 h with CSE 500 or 750 µg/ml or vehicle control (DMSO), stained with FerroOrange, and quantified by 3D-ARQ. A statistically significant increase in intracellular iron accumulation (Fe^2+^) was observed in CSE-treated ROs compared to controls. DMSO = 9277.600 ± 465.89 (*n* = 10), CSE-500µg/ml=16200.400 ± 902.77 (*n* = 10) and CSE-750 µg/ml=14849.400 ± 958.92 (*n* = 10). Error bars represent Mean ± SEM; one-way ANOVA; ^****^p < 0.0001. **B** Staining of CSE- or vehicle (DMSO) control-treated ROs with BODIPY C11 581/591 assessed longitudinally at 0, 6, 24, 48 and 72 h showed statistically significant increases in the ratios of peroxidized/ non-peroxidized lipids in CSE-treated ROs compared to controls. Error bars represent Mean ± SEM; *n* = 10 ROs per condition. **C** ROs treated with CSE or vehicle (DMSO) control were lysed, and reduced glutathione (GSH) was detected using a fluorescence assay. Bar graph shows a statistically significant increase in GSH fluorescent intensity in CSE-treated ROs compared to DMSO. DMSO = 1347.737 ± 99.16 (*n* = 3), CSE-500µg/ml=1765.088 ± 78.65 (*n* = 3), and CSE-750 µg/ml=1963.369 ± 89.00 (*n* = 3). Error bar shows Mean ± SEM; One way ANOVA; ^*^p < 0.04; ^**^p < 0.007. **D** Oxidized glutathione (GSSG) was quantified in RO lysates using a fluorescence-based assay. A statistically significant increase in GSSG was detected in CSE-treated ROs compared to vehicle controls (DMSO). DMSO = 3799.963 ± 73.77 (*n* = 3), CSE-500µg/ml=6310.966 ± 193.15 (*n* = 3), and CSE-750 µg/ml=10609.686 ± 653.896 (*n* = 3). Error bar shows Mean ± SEM; One way ANOVA; ^**^p < 0.001 ^****^p < 0.0001. **E** The GSH/GSSG ratio was significantly decreased in CSE-treated ROs compared to vehicle-treated controls (DMSO), indicating an oxidative cellular environment. DMSO = 0.354 ± 0.02 (*n* = 3), CSE-500µg/ml=0.279 ± 0.01 (*n* = 3), and CSE-750 µg/ml=0.186 ± 0.01 (*n* = 3). Error bar shows Mean ± SEM; One way ANOVA; ^*^p < 0.04; ^***^p < 0.0007.

Growing evidence indicates that ferroptosis, a newly identified form of regulated cell death marked by iron-dependent accumulation of lipid peroxides, is an additional contributor to the pathogenesis of AMD [52]. To investigate whether CSE induces ferroptosis in human ROs, we measured intracellular iron (Fe^2+^) accumulation in control- and CSE-treated live ROs by 3D-ARQ using the FerroOrange fluorescent dye. We observed that CSE-treated ROs at both tested concentrations exhibited significantly increased ferrous ion accumulation compared to vehicle-treated ROs (Fig. 3A). Since iron accumulation leads to lipid peroxidation via the Fenton reaction [53], we investigated the levels of lipid peroxides under our experimental and control conditions using the BODIPY C11 581/591 fluorescence-based assay. In this assay, oxidation of the polyunsaturated butadienyl portion of fatty acid analogs causes a shift in fluorescence emission from red (~590 nm) to green (~510 nm). Thus, the red-to-green ratio is a direct measure of lipid oxidation and free radical formation. Our results showed a significant increase in the BODIPY C11 fluorescent ratio in CSE-treated ROs after 6, 24, 48, and 72 h of treatment, compared to vehicle-treated controls (Fig. 3B).

Finally, we went on to evaluate the effects of these processes on the cellular antioxidant defense. Glutathione plays a crucial role in protecting retinal cells from oxidative stress and ferroptosis. Serum from AMD patients has decreased ratios of reduced to oxidized glutathione (GSH/GSSG), indicative of a weaker antioxidant defense system that contributes to the progression of AMD [54]. We thus measured GSH, GSSG, and the GSH/GSSG ratio using a colorimetric assay. Notably, we observed a significant increase in both GSH (Fig. 3C) and GSSG levels upon CSE treatment (Fig. 3D), which may indicate an increase in glutathione synthesis in response to cellular stress. However, the GSH/GSSG ratio was reduced (Fig. 3E), suggesting a shift in the redox balance towards an oxidative cellular environment.

Overall, these results support the hypothesis that, upon CSE treatment of ROs, an iron overload exacerbates oxidative stress, contributing to lipid peroxidation and a perturbed redox balance, thus triggering ferroptotic cell death as observed in AMD patients.

### Proteomic analysis elucidates molecular mechanisms involved in CSE-induced cell death pathways

**Fig. 4:**
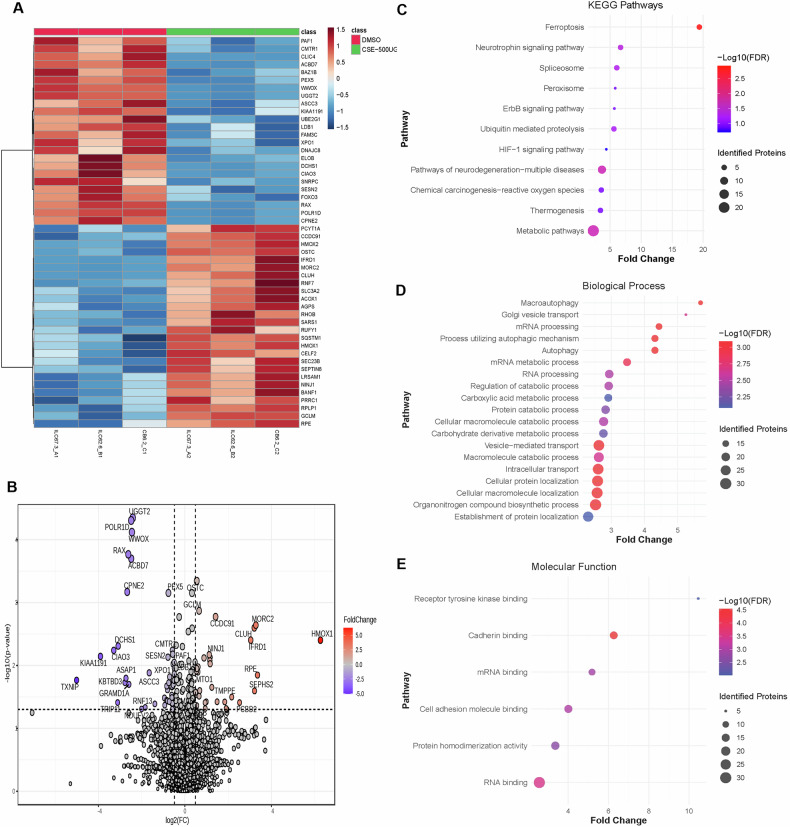
Quantitative proteomic analysis identifies CSE-induced cellular and molecular mechanisms of retinal degeneration. **A** Protein was extracted from ROs treated with CSE 500 µg/ml or vehicle control (DMSO) and subjected to proteomic analysis by LC-MS. Graph represents heatmap and hierarchical clustering of the top 50 differentially expressed proteins. Each column represents an independent hiPSC line (ILC67.3, ILC62.6, and CB6.2). **B** Volcano plot representation of differentially expressed proteins (DEPs). Direction of comparison: CSE_500µg/DMSO-treated controls. Log2(FC) indicates Fold Change; threshold: 1.4; *p* < 0.05; 80 significant DEPs were identified. Black dots represent no significant change, blue dots represent down-regulated, and red dots represent up-regulated DEPs. **C** KEGG pathway enrichment analysis was performed to identify the biological pathways significantly associated with DEPs in CSE-500 µg/ml treated ROs compared to DMSO-treated controls. **D** Biological process analysis was performed to identify the pathways significantly associated with DEPs in CSE-500 µg/ml-treated ROs compared to vehicle control (DMSO). **E** Molecular Function analysis was performed to identify the pathways significantly associated with DEPs in CSE-500 µg/ml treated ROs compared to vehicle control (DMSO).

To further investigate the molecular mechanisms triggered by CSE that induce retinal degeneration in ROs, we performed differential proteomic analyzes on CSE-treated ROs and vehicle-treated controls using liquid chromatography-mass spectrometry (LC-MS). The data from this analysis is available at ProteomeXchange, with identifier PXD063075. A total of 5078 proteins were identified and quantified at FDR < 1%. Statistically significant differences were observed in 159 differentially expressed proteins (DEPs) between CSE-500 µg/ml- and vehicle-treated ROs (Supplementary File 2). Hierarchical clustering was performed to assess global protein expression patterns and to identify the distinct proteomic signatures between these conditions. The heatmap in Fig. 4A represents the top 50 DEPs. Protein abundance levels were standardized and represented as a comparison between DMSO and CSE-500 µg/ml from different cell lines. Notably, PAF1, CLCI4, ACBD7, BAZ1B, FAM3C, FOXO3, HMOX2, HMOX1, SLC3A2, RHOB, GCLM, and RPE were significantly dysregulated in CSE-treated clusters compared to DMSO. These proteins are associated with various metabolic and cellular processes, including cell survival, apoptosis, DNA damage, maintaining RPE homeostasis, eye development and function, oxidative stress, and the production of glutathione [55–61]. A volcano plot generated with a statistical significance threshold of *p* < 0.05 identified a total of 80 DEPs (Fig. 4B).

To gain further insight into the pathophysiological mechanisms induced in this model, we performed gene ontology analysis on our proteomics dataset. KEGG pathway analysis (Fig. 4C) and bioinformatic analysis of predicted cellular components (Fig. 4D), molecular functions (Fig. 4E) and biological processes (Fig. 4F) revealed significant associations of DEPs with multiple pathways involved in AMD pathophysiology. Among the top enriched pathways, ferroptosis showed the highest fold enrichment (-log10 fold enrichment > 20, FDR < 0.01). Among the DEPs associated with the ferroptosis pathway in our dataset were GCLM, HMOX1, and SLC3A2, which were highly upregulated, and ACSL3 and MAP1LC3A, which were downregulated. Metabolic pathways showed the second-highest fold enrichment in KEGG analysis (Fig. 4C), suggesting metabolic dysregulation in disease progression, impaired protein synthesis, and mitochondrial dysfunction. These included enrichment in biological processes such as macroautophagy, autophagy, and catabolic activity, suggestive of a neurodegenerative process (Fig. 4F). Bioinformatic analysis of the molecular function pathways revealed dysregulation of proteins that interact with receptor tyrosine kinase, cadherin, and cell adhesion molecules, which disrupt signaling mechanisms related to cell survival, growth, inflammation, and structural integrity, RNA binding, and protein homodimerization, and which are important for gene expression regulation, protein-protein interactions, and the cellular stress response (Fig. 4E).

Analysis of DEPs between vehicle-treated control and CSE 750 µg/ml-treated ROs showed similar results (Fig. S4A–E), suggesting that increased concentration of CSE results in increased neurodegeneration via similar molecular mechanisms.

Overall, our proteomic analysis revealed that DEPs are significantly enriched in pathways associated with AMD pathogenesis, including oxidative stress, iron regulation, lipid peroxidation, inflammation, mitochondrial dysfunction, retinal structure and integrity, and neurodegenerative diseases.

## Discussion

Photoreceptor loss in dry AMD is a complex process that results in irreversible vision loss, constituting a major public health concern [62]. Currently, effective treatments are lacking. Taking advantage of state-of-the-art human retinal organoid technologies, we developed a new model of photoreceptor cell death that mimics aspects of dry AMD pathophysiology and that can be used for drug screening and hit validation.

Photoreceptor damage associated with dry AMD is driven by oxidative damage, mitochondrial dysfunction, and disrupted cellular homeostasis [44, 63–65]. Previous studies have reported that in human AMD, RPE, photoreceptors, and inner nuclear layer cells die by apoptosis [66, 67]. Even though studies investigating the molecular mechanisms leading to photoreceptor apoptosis in human AMD are scarce, with only one study looking at increases in Fas [12], it is well established that damage to the mitochondria due to oxidative stress can lead to the activation of the intrinsic apoptotic pathway [12, 49, 68]. Additionally, aging, the main risk factor in AMD, leads to increased iron levels in the retina, and iron balance dysregulation can contribute to the pathogenesis of AMD by exacerbating oxidative damage and inducing cell death via ferroptosis [69–71].

Cigarette smoking is also an important environmental risk factor for AMD, as well as a potent oxidative stressor [2, 35, 72]. Thus, cigarette smoke and its extract have been used to develop RPE cell culture and animal models of AMD-like pathology [35, 73–75]. In this study, we show that CSE treatment of human ROs can mimic photoreceptor cell death in dry AMD. Our results revealed an increase in phosphatidylserine externalization and DNA fragmentation, as well as caspase-3 and caspase-9 activation in CSE-treated ROs compared to controls, suggesting the involvement of the intrinsic apoptotic pathway. This was also supported by our MILIPLEX and proteomics studies showing a significant increase in the BAD/BCL2 ratio. The TUNEL- and activated caspase-positive cells induced by CSE treatment were mainly co-localized with photoreceptor markers NRL, RXR-y, and recoverin, though some inner retinal cells were also affected, as is the case in human AMD retinas [76].

CSE treatment also increased ROS production and mitochondrial membrane depolarization, in line with observations in human AMD tissues as well as animal models of AMD [77–79]. This increase in oxidative damage eventually overwhelms the cellular antioxidant defenses, as evidenced by the decrease in reduced to oxidized glutathione ratios. Ferroptosis, a cell death mechanism in which the accumulation of iron causes lipid peroxidation and the generation of ROS, can compound these effects, leading to increased cellular damage [80]. We found that CSE treatment of ROs causes an increase in intracellular iron and lipid peroxidation. Additionally, our proteomic analysis identified many DEPs associated with ferroptosis, including the iron-regulating proteins heme-oxygenase 1 and 2 (HMOX1 and HMOX2), which have been associated with neuronal damage and retinal degeneration by sensing the increased oxidative stress [58]. Together, these findings indicate that iron overload exacerbates ROS production and lipid peroxidation, further contributing to photoreceptor cell death in the CSE-RO model.

Moreover, proteomic analysis provided further mechanistic insights, highlighting an enrichment in DEPs related to metabolic dysregulation, impaired protein synthesis, autophagy, and overall neurodegeneration. For example, we identified a significant decrease in FOXO3, which acts as an antioxidant and anti-apoptotic and is crucial in regulating the aging process by influencing cell cycle arrest, autophagy, and DNA repair in multiple ocular cells, and in maintaining photoreceptor integrity [55]. We also found a significant decrease in CLIC4, a multifunctional protein that regulates diverse biological processes and whose deficit leads to AMD-like phenotypes in animal models [61, 81]. Together, these findings underscore the potential of this model not only for therapeutic development but also for mechanistic studies and for the discovery of novel biomarkers for dry AMD.

Despite its significant advantages, this model is not without limitations. These include those that are characteristic of ROs, such as the absence of a fovea. Additionally, the retinal vasculature and microglia are also absent, and thus, retinal organoids cannot fully recapitulate the inflammatory landscape of the disease. Another important consideration is the lack of photoreceptor-RPE juxtaposition, which must be taken into account when modeling dry AMD, a disease in which RPE damage and death contribute significantly to photoreceptor degeneration. While RPE culture models exist and have been used for decades to investigate aspects of AMD pathophysiology and evaluate potential treatments, complementary models of photoreceptor degeneration are largely lacking. Thus, we aim to fill this niche by introducing the first human-RO model that mimics the pathophysiological mechanisms affecting photoreceptors in dry AMD. This has important implications for drug development applications, as it enables the evaluation of photoreceptor-protective therapies in a human context. Considering that currently approved treatments have been shown to delay the progression of the RPE lesion but have not provided improvements in visual function, a photoreceptor protective strategy may be necessary to complement these treatments and provide a more effective therapeutic approach. Additionally, multiple RPE transplant products are currently in phase I/II clinical trials [82, 83]. This is an exciting new approach for dry AMD treatment, but the success of these strategies depends on the presence of viable photoreceptor cells, as they would not provide benefit to an already degenerated outer retina. Thus, the use of this model to evaluate photoreceptor protection in a dry AMD environment could improve the success of these novel therapeutics.

An additional consideration is that CSE is a complex chemical mixture that can elicit a mix of cellular stress processes. Since standardization of the product is important for reproducibility, we have used a well-standardized, commercially available product and evaluated our main outcomes using multiple lots with identical results. However, we also provide an initial characterization of two alternative approaches: H_2_O_2_ and NaIO_3_ treatment. These offer the advantage of being chemically defined and inducing a narrower range of intracellular mechanisms. While these may be preferable for some applications, AMD is a complex disease where multiple cellular mechanisms overlap to cause photoreceptor degeneration, and thus, a simplified approach also runs the risk of not adequately mimicking the disease conditions and leading to the advancement of drugs that may be ineffective in clinical trials. Thus, we believe our CSE-based paradigm may be better suited for these scenarios.

In the future, it may be valuable to develop RPE-RO co-culture models, as well as co-cultures that integrate microglia, or the development of vascularized systems.

Finally, another important outcome of this work is the establishment of a suite of fluorescence-based, quantitative outcome measures in live retinal organoids. These assays enable real-time, multiparametric analysis of key disease processes such as cell death, mitochondrial function, oxidative stress, lipid peroxidation, and others, in a nondestructive, scalable, and reproducible manner. The combination of this model and technologies provides a novel translational platform for elucidating dry AMD mechanisms and testing potential therapeutics in a controlled, human-specific context. As proof-of-concept of the value of this platform for drug development, we have recently used this model to evaluate the neuroprotective efficacy of H105A, a pigment epithelium-derived factor-derived peptide, in photoreceptor degeneration [84].

Given the complexity of AMD, a combination of models, including organoids, RPE cultures, animal systems, and clinical tissues, may be necessary to achieve a comprehensive insight into the disease and accelerate the development of effective treatments.

## Materials and methods

### Human-induced pluripotent stem cell lines and RO generation

We utilized three human-derived induced pluripotent cell lines: A18945 (Gibco), ILC67.3, and ILC42.3 (both obtained from the Gates Institute at CU Anschutz), sourced from female cord blood, female urine-derived renal epithelial cells, and male urine-derived renal epithelial cells, respectively. All cell lines were karyotyped and regularly tested for mycoplasma contamination using the Mycoplasma PCR detection kit (Abcam, #ab289834). The use of hiPSCs in this study conforms to the University of Colorado Institutional Biosafety Committee (IBC). hiPSCs were cultured on Matrigel (Corning #354230) coated plates and maintained up to 70% confluency until ready for differentiation in mTeSR1 media (StemCell Technologies #85850).

We followed the Zhong et al. [24] protocol to differentiate the hiPSCs into ROs. Briefly, on day 0, hiPSC colonies were dissociated with dispase, grown in suspension to form embryoid bodies (EBs), and slowly transitioned to media containing DMEM/F12 (1:1), 1% N2 supplement (Invitrogen), 1× minimum essential media-nonessential amino acids (NEAAs), 2 µg/ml heparin (Sigma). On day 7, EBs were seeded on Matrigel-coated plates, and on day 16, the media was switched to DMEM/F12 (3:1), 2% B27 (without vitamin A, Invitrogen), 1x NEAA, and 1% antibiotic–antimycotic (Gibco). Retinal domains were identified and lifted manually with tungsten needles between days 21 and 26 under a phase contrast microscope. On day 30, the culture medium was transitioned to DMEM/F12 (3:1) with 2% B27, 1× NEAA, 1% antibiotic–antimycotic, 10% fetal bovine serum (FBS; Gibco), 100 mM Taurine (Sigma), and 2 mM GlutaMAX (Invitrogen). Additionally, 1 µM retinoic acid supplement was introduced daily starting from day 63. On day 91, the medium was changed to DMEM/F12 (1:1) supplemented with 1% N2, 1× NEAAs, 1% antibiotic–antimycotic, 10%FBS, 100 mM Taurine and 2 mM GlutaMAX, and the concentration of retinoic acid supplementation was reduced to 0.5 µM for the remainder of the culture period. The use of hiPSCs in this study conforms to the University of Colorado Institutional Biosafety Committee standards.

### Cigarette smoke extract, hydrogen peroxide, and sodium iodate treatment of ROs

CSE was purchased from Murthy Pharmaceuticals, Lexington, KY, USA (Catalog #NC1560725). CSE contains 40 mg/ml condensate and 6% nicotine, and was prepared by smoking University of Kentucky’s 1R6F Standard Research Cigarettes on an FTC smoke machine. Total particulate matter on the filter was calculated by the weight gain of the filter after smoking. The amount of DMSO was calculated to produce a 4% (40 mg/ml) solution. The extract was diluted in the medium used for the treatment. On day 180 of differentiation, ROs were treated with 500, 750, or 1000 µg/ml of CSE or vehicle control (DMSO) for 48 h or 5 days at 37 °C in a CO_2_ incubator.

Alternatively, ROs were treated with H_2_O_2_ at a 1 mM concentration or with NaIO_3_ at 0.5 mM in media, and cultured for 48 h at 37 °C in a CO_2_ incubator.

### Fluorescence-based quantification of outcome measures in live ROs

ROs were plated individually into each well of transparent U-bottom 96-well plates (NUNC, Rochester, NY, USA Catalog #4515). ROs were washed three times with buffer, as explained below for each assay. Clear media was used for background reading. Auto-fluorescent background reading was measured in non-stained ROs by 3D-ARQ as described in Vergara et al. [37], using a Tecan Spark microplate reader and SparkControl v2.3 software. The Z-position of ROs was measured by manually selecting each well with optimized parameters and modes for different assays. Data analysis was performed as described in Vergara et al. [37].

### Assessment of mitochondrial health using JC-1 dye in live ROs

ROs were plated in a 96-well plate with clear media, and the background fluorescence of individual ROs was assessed by 3D-ARQ. ROs were then treated with 4.6 µM JC-1 dye (ThermoFisher Scientific, Waltham, MA, USA Catalog #T3168) for 1 h at 37 °C, followed by 3 washes in 1X-DPBS. Fluorescence readouts were obtained using the following parameters: fluorescent top mode plate reading; Wavelength for green shift: Excitation: Emission 485:530 nm, Excitation bandwidth: 10 nm, Emission bandwidth: 15 nm; Wavelength for redshift: Excitation: Emission 535:590 nm, Excitation bandwidth: 15 nm, Emission bandwidth: 15 nm; Gain: 120, Manual; Number of flashes: 50; integration time: 40 µs; lag time 0 µs. ROs were then treated with CSE or DMSO as described above, and longitudinal readings were measured at 6, 24, 48, and 72 h after treatment. Results were analyzed after background subtraction. We used *N* = 10 ROs for each experimental condition and cell line.

### Measurement of reactive oxygen species (ROS) in live ROs

ROs were treated with vehicle control or CSE as described above, or with Antimycin-A (10 µg, abcam#ab236206), as a positive control for 48 h, then treated with 5 µM dihydroethidium (DHE) for 45 min at 37 °C in a CO_2_ incubator. They were then washed three times in cell-based assay buffer, placed in clear media, and evaluated by 3D-ARQ. The optimized parameters were: fluorescent top reading mode, Wavelength: Excitation: Emission 520:600 nm, Excitation bandwidth: 5 nm, Emission bandwidth: 10 nm; Gain: 120, Number of flashes: 30; integration time: 40 µs; lag time 0 µs. Results were analyzed after background subtraction. *N* = 10 ROs for each experimental condition and cell line.

### Cell death assessment in live ROs

ROs were incubated with 10 µM PSVue-794 (Molecular Target Technologies, P-1001) or 4 µM ethidium homodimer (EtHD) (ThermoFisher Scientific, L3224) for 1 h and 45 min at 37 °C, washed three times with 1X-TES buffer and D-PBS, placed in clear media, and analyzed by 3D-ARQ. Optimized parameters for PSVue were Excitation: Emission: 730:820 nm, Excitation bandwidth: 10 nm, Emission bandwidth: 15 nm; Wavelength for EtHD: Excitation: Emission 530:630 nm, Excitation bandwidth: 5 nm, Emission bandwidth: 5 nm; Gain: 160, Manual; Number of flashes: 20; integration time: 40 µs; lag time 0 µs was used with top mode reading for measurement. Results were analyzed after background subtraction; *N* = 10 ROs per condition and cell line.

### Lipid peroxidation assessment in live ROs

After CSE and vehicle control treatment, ROs were treated with 10 µM BODIPY 581/591-C11 (Invitrogen #D3861) dye for 1 h at 37 °C, to measure the oxidized phospholipid as a marker of lipid peroxidation inside the cells. After washing with cell-based assay buffer and plating with the same buffer as recommended by the manufacturer, they were analyzed by 3D-ARQ following with the following parameters: fluorescent top reading mode; Wavelength for green shift: Excitation: Emission 485:530 nm, Excitation bandwidth: 10 nm, Emission bandwidth: 15 nm; Wavelength for redshift: Excitation: Emission 535:600 nm, Excitation bandwidth: 15 nm, Emission bandwidth: 15 nm; Gain: 120, Manual; Number of flashes: 50; integration time: 40 µs; lag time 0 µs. Results were analyzed after background subtraction; *N* = 10 ROs per condition and cell line.

### Detection of Intracellular iron (Fe2+) in live ROs

ROs treated with CSE or DMSO were incubated with 1 µM ferroOrange dye (DojinDo Laboratories, Mashikimachi, Japan, Catalog #F374) in HBSS for 45 min at 37 °C. Fluorescence intensity was read in the same buffer without washing, as per the manufacturer’s protocol. 3D-ARQ parameters were: Fluorescent top reading mode; Wavelength: Excitation: Emission 500:590 nm, Excitation bandwidth: 5 nm, Emission bandwidth: 10 nm; Gain: 140, Number of flashes: 30; integration time: 40 µs; lag time 0 µs. Results were analyzed after background subtraction; *N* = 10 ROs per condition and cell line.

### GSH/GSSG ratio detection in RO lysates

ROs were treated as specified, and cellular GSH levels were measured using the GSH/GSSG ratio detection assay kit (Abcam, Cambridge, MA, USA. Catalog #ab138881) according to the manufacturer’s detailed protocols. Briefly, the GSH assay mixture was added to the RO lysate for a one-step fluorometric reaction and incubated for 60 min at room temperature (RT), protected from the light. Fluorescence intensity was measured using a Tecan Spark plate reader and SparkControl v2.3 software at the excitation/emission wavelength of 490/520 nm. GSH, GSSG levels, and GSH/GSSG ratios were calculated using a standard curve. Results were analyzed after background subtraction; *N* = 6 ROs per condition and cell line.

### TUNEL and immunofluorescence staining

After treatment with experimental conditions (*N* = 5 for each condition), ROs were washed with 1X-PBS three times and fixed with 4% paraformaldehyde for 10 min at RT. ROs were then sequentially incubated in 6.75%, 12.5%, and 25% sucrose solutions in 1X-PBS, embedded in OCT:Sucrose (1:1) solution, and stored at −80 °C. 10 μm cryosections were prepared using a Leica Cryostat. Slides were dried and washed with 1X PBS for 15 min, rinsed three times with DI water for 5 min and incubated in 1× Terminal Transferase (TdT) buffer containing 1X buffer 4 (NEB) and 2.5 μM CoCl_2_ for 10 min at RT in a humid chamber. For labeling, 1XTdT buffer, biotinylated 16-dUTP (30 µM), and 1X-Terminal transferase were added to each slide and incubated for 1 h at 37 °C in a humid chamber. The reaction was stopped by incubating slides in 2XSSC for 15 min at RT, followed by 1X PBS wash. Blocking was performed using 2% Bovine serum albumin (BSA) in 1X PBS for 10 min at RT, and slides were incubated with streptavidin CY3 (10 µg/ml) for 45 min at RT in a humid chamber. Subsequently, slides were washed 3 times with 1X PBS, blocked with 5% BSA and 10% normal donkey serum (DS) in 1X-PBS with 0.50% Triton X-100 for 30 min at RT in a humid chamber and then incubated overnight with primary antibodies listed in supplementary table 1 diluted in 1% BSA and 2% DS in 1X-PBS with 0.50% Triton X-100 at 4 °C. Subsequently, slides were washed three times with 1X-PBS with 0.25 Triton X-100 and incubated with secondary antibodies listed in Supplementary Table 2, diluted (1/3000) in 1% BSA and 2% DS in 1X-PBS with 0.5% Triton X-100 at RT for 2 h, protected from light. Slides were washed for 5 min 3 times with 1X-PBS with 0.25% Triton X-100, counterstained with DAPI for 10 min, washed with 1X PBS and DI water, and mounted using FluoroMount solution.

### Image analysis and quantification

Fluorescence images were acquired at 20× and 40× with a Nikon AXR and C2 Confocal Microscope A1 (Minato City, Tokyo, Japan) and stitched together using Nikon Elements Software. Images were analyzed using Fiji software (Bethesda, MD, USA). At least five retinal organoids were analyzed per assay for each cell line. The relative area of positive immunostaining was calculated for each marker as a percentage of the total retinal area defined by DAPI. Colocalization analysis of cell death and photoreceptor markers was conducted by measuring the double-labeled area and by counting the cells positive for specific markers with Fiji and expressing it as a percentage of the total photoreceptor-positive area.

### MILLIPLEX apoptosis assay

ROs were lysed with MILLIPLEX lysis buffer (1X) (Millipore #43-040) provided in the kit, with 1X protease inhibitor (Sigma, Saint Louis, MO, USA, Catalog #P8340). Protein was quantified using Pierce™ BCA Protein Assay Kit (ThermoFisher Scientific; Waltham, MA, Catalog #23225). Apoptosis signaling molecules were detected using the MILLIPLEX Early Phase Apoptosis 7-plex signaling kit (Millipore; Burlington, MA, USA, Catalog #48-669mag) by following the manufacturer’s detailed protocol, and median fluorescence intensity was measured using a BioRad Bioplex200 instrument. Results were analyzed manually after blank subtraction using Microsoft Excel; graphs were plotted using GraphPad (*N* = 10 for each cell line/experimental condition).

### Proteomics

Samples were reduced, alkylated, and digested using S-TrapTM micro filters (Protifi, Huntington, NY) according to the manufacturer’s protocol. Digested peptides were cleaned using PierceTM C18 Spin Tips (Thermo Scientific) according to the manufacturer’s protocol, dried in a vacuum centrifuge, and resuspended in 0.1% formic acid in mass spectrometry-grade water. Digested peptides were loaded onto individual Evotips following the manufacturer’s protocol and separated on an Evosep One chromatography system (Evosep, Odense, Denmark) using a Pepsep column (150 μm inner diameter, 15 cm) packed with ReproSil C18 1.9 μm, 120 A resin. The system was coupled to the timsTOF Pro mass spectrometer (Bruker Daltonics, Bremen, Germany) via the nano-electrospray ion source (Captive Spray, Bruker Daltonics). The mass spectrometer was operated in PASEF mode. The ramp time was set to 100 ms, and 10 PASEF MS/MS scans per topN acquisition cycle were acquired. MS and MS/MS spectra were recorded from *m*/*z* 100 to 1700. The ion mobility was scanned from 0.7 to 1.50 Vs/cm^2^. Precursors for data-dependent acquisition were isolated within ±1 Th and fragmented with an ion mobility-dependent collision energy, which was linearly increased from 20 to 59 eV in positive mode. Low-abundance precursor ions with an intensity above a threshold of 500 counts but below a target value of 20,000 counts were repeatedly scheduled and otherwise dynamically excluded for 0.4 min.

Fragmentation spectra were searched against the UniProt human proteome database using the MSFragger-based FragPipe computational platform [85]. Contaminants and reverse decoys were added to the database automatically. The precursor-ion mass tolerance and fragment-ion mass tolerance were set to 15 and 20 ppm, respectively. Fixed modifications were set as carbamidomethyl (C), and variable modifications were set as oxidation (M), two missed tryptic cleavages were allowed, and the protein-level false discovery rate (FDR) was ≤1%. The mass spectrometry proteomics data have been deposited to the ProteomeXchange Consortium via the PRIDE partner repository with the dataset identifier PXD063075.

### Statistical analysis

Retinal organoid differentiation was performed 5–6 times to obtain enough biological replicates for different experiments. Proteomic experiment analysis was conducted on all three hiPSC lines. All other experiments were repeated with two independent hiPSC lines, except for the studies on iron accumulation, lipid peroxidation, and the glutathione levels. The number of independent biological replicates is detailed in the figure legends for each experiment. In live ROs experiments, ROs were randomly assigned to different groups and plated individually in wells of 96-well plates. Analysis of live assays was automated to prevent human bias, as previously described [37]. ROs that were damaged during staining or washes were excluded from the analysis. Statistical analysis was conducted using GraphPad Prism 10.10 software versions, employing an Unpaired Student’s *t*-test and one-way ANOVA with a Tukey post-hoc test for multiple comparisons after normality assessment. The bar and line graphs display the mean ± standard error of the mean with individual values as plotted. Asterisks were used to represent significant differences (^*^p < 0.05; ^**^p < 0.005; ^***^p < 0.0002; ^****^p < 0.0001).

### Ethics approval and consent to participate

All experimental procedures were conducted in accordance with relevant institutional guidelines and regulations. Human iPSC lines used in this study were obtained from established sources and handled in compliance with the University of Colorado Institutional Biosafety Committee (IBC) regulations and approvals. No human participants were directly involved in this study, and no identifiable human data were collected. Therefore, institutional review board (IRB) approval and informed consent to participate were not required. The providers of the hiPSC lines obtained the materials under informed consent and IBR approval, and our work complies with the MTA agreements with those sources.

## Supplementary information

Supplementary file 1. Supplemental figures and tables

Supplemental file 2. List of DEPs

## Data Availability

The mass spectrometry proteomics data generated in this study have been deposited to the ProteomeXchange Consortium via the PRIDE partner repository under the dataset identifier PXD063075. All other data supporting the findings of this study are available from the corresponding author upon reasonable request.
