# Supplementary material for: Human retinal organoid model of disease-relevant photoreceptor cell death amenable to drug screening
Source: Cell Death Dis. 2026 Apr 13;17(1):474. doi: 10.1038/s41419-026-08724-y (PMC13184248; doi:10.1038/s41419-026-08724-y)
Supplement: Supplementary file 1 — Supplemental figures and tables [file 41419_2026_8724_MOESM1_ESM.docx]

**Supplementary Information**

**Human Retinal Organoid Model of Disease-Relevant Photoreceptor Cell Death Amenable to Drug Screening**

Shama Parween, Anthony J. Saviola, Anna C. Howell, Stefanie Varghese, David Ceja Galindo, and M. Natalia Vergara

**Contents:**

- Figure S1 and legend: Schematic representation of retinal organoid generation from human iPSCs.
- Figure S2 and legend: Apoptosis induction in ROs treated with CSE for five days.
- Figure S3 and legend: Validation of H_2_O_2_ and NaIO_3_-induced cellular stress in retinal organoids
- Figure S4 and legend: Proteomic analysis of ROs treated with CSE-750 µg/ml or DMSO for 48 h.
- Tables S1 and S2: Lists of primary and secondary antibodies used for immunofluorescence studies.

**Figure S1. Schematic representation of retinal organoid generation from human iPSCs**


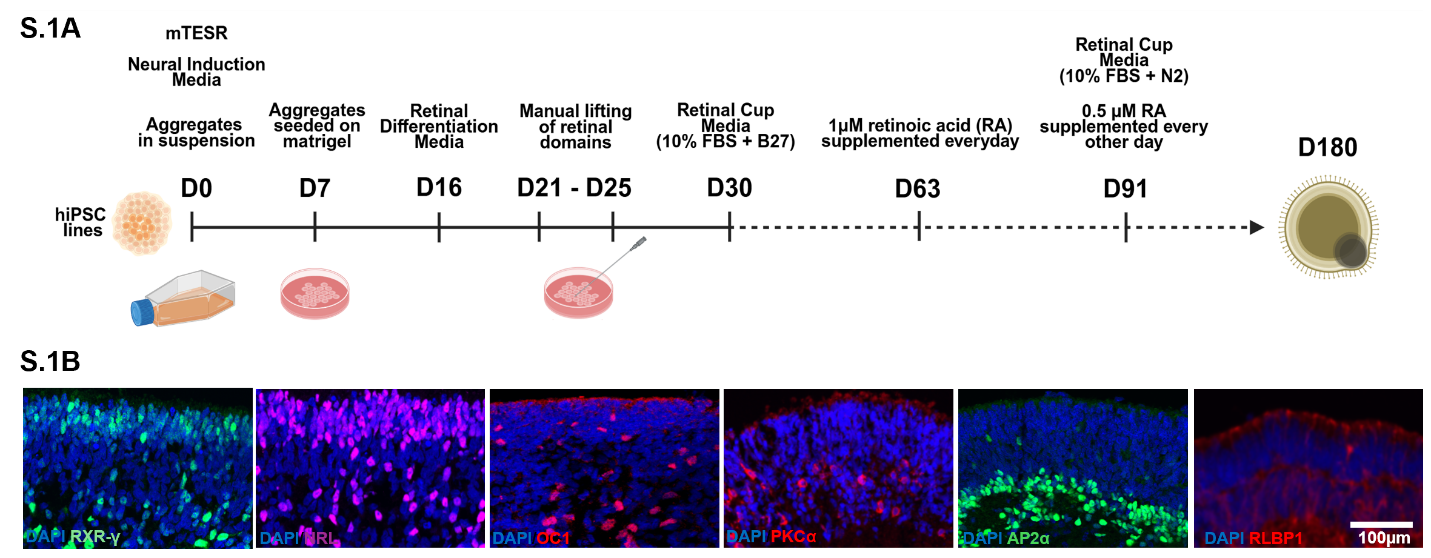


1. Human-induced pluripotent stem cells (iPSCs) were differentiated through a stepwise process into neuroectodermal progenitors, followed by optic vesicle–like structures that mature into laminated, self-organizing retinal organoids containing the major retinal cell types (See Methods and Zhong et al., 2014).
2. Immunofluorescent staining of RO at 180 days of differentiation demonstrated the laminated retinal structure and presence of the main retinal cell types within stem cell-derived organoids. Cone photoreceptors (RXR-γ), rod photoreceptors (NRL), horizontal cells (OC1), bipolar cells (PKCα), amacrine cells (AP2α), and Müller glia (RLBP1) were observed in distinct layers, closely resembling the laminar structure of the native retina. Scale bar: 100µm.

**Figure S2. Apoptosis induction in ROs treated with CSE for five days.**

**
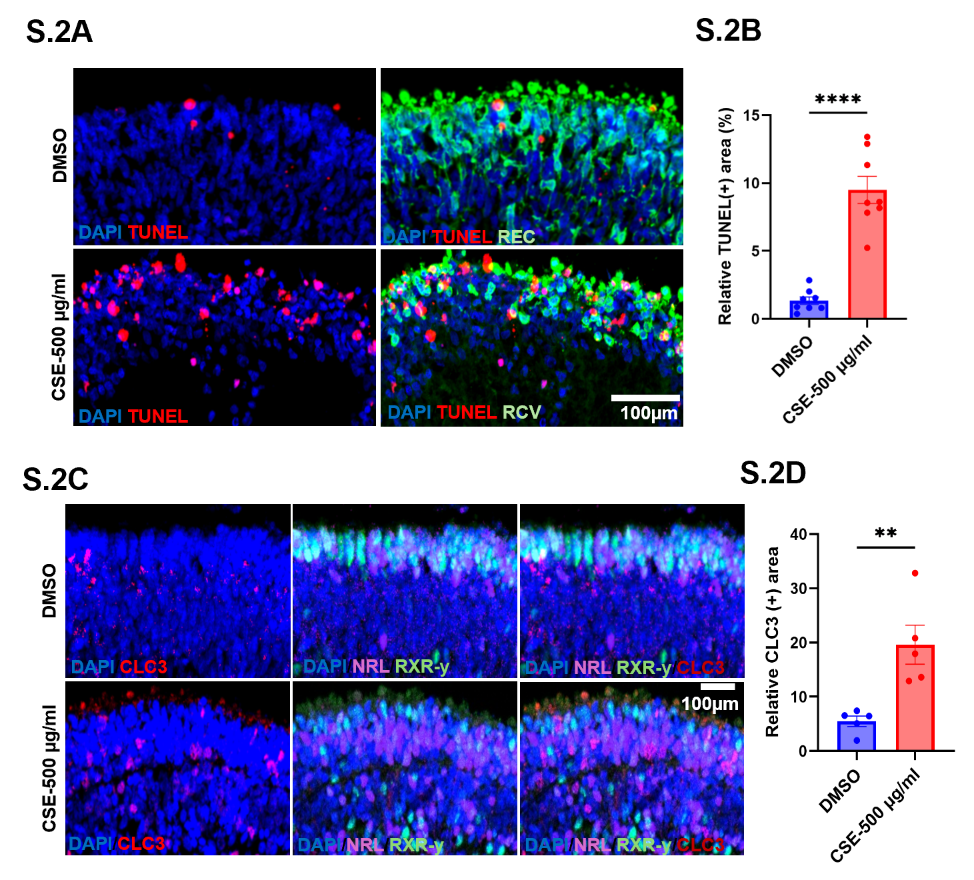
**

ROs were treated with vehicle control (DMSO) or CSE-500 µg/ml for 5 days to evaluate the extent of apoptotic cell death:

1. Representative confocal micrographs of D180 RO sections stained with TUNEL to examine DNA fragmentation, along with recoverin (RCV) staining to identify photoreceptor cells. Scale bar: 100µm.
2. TUNEL staining quantification shows a statistically significant increase in cell death in CSE-treated ROs compared to vehicle controls (DMSO). Relative TUNEL (+) area: DMSO: 1.342±0.28 (n=8), and CSE-500 µg/ml: 9.501±0.988 (n=8). Error bars indicate Mean±SEM; T-test; ****p<0.0001.
3. Immunofluorescence staining was performed on D180 RO sections using antibodies for CLC3 and photoreceptor markers NRL (Rod) and RXR-γ (Cone). Colocalization of CLC3 and photoreceptor markers was also analyzed. Scale bar: 100µm.
4. Bar graph represents the quantification of relative CLC-3-labeled area/DAPI. A significant increase in CLC3-mediated photoreceptor cell death was observed in CSE-treated ROs compared to vehicle controls (DMSO). DMSO= 5.483±0.95 (n=5), and CSE-500 µg/ml= 19.622±3.599 (n=5). Error bar shows Mean±SEM; T-test; **p<0.005.

**Figure S3. Validation of H_2_O_2_ and NaIO_3_-induced cellular stress in retinal organoids.**


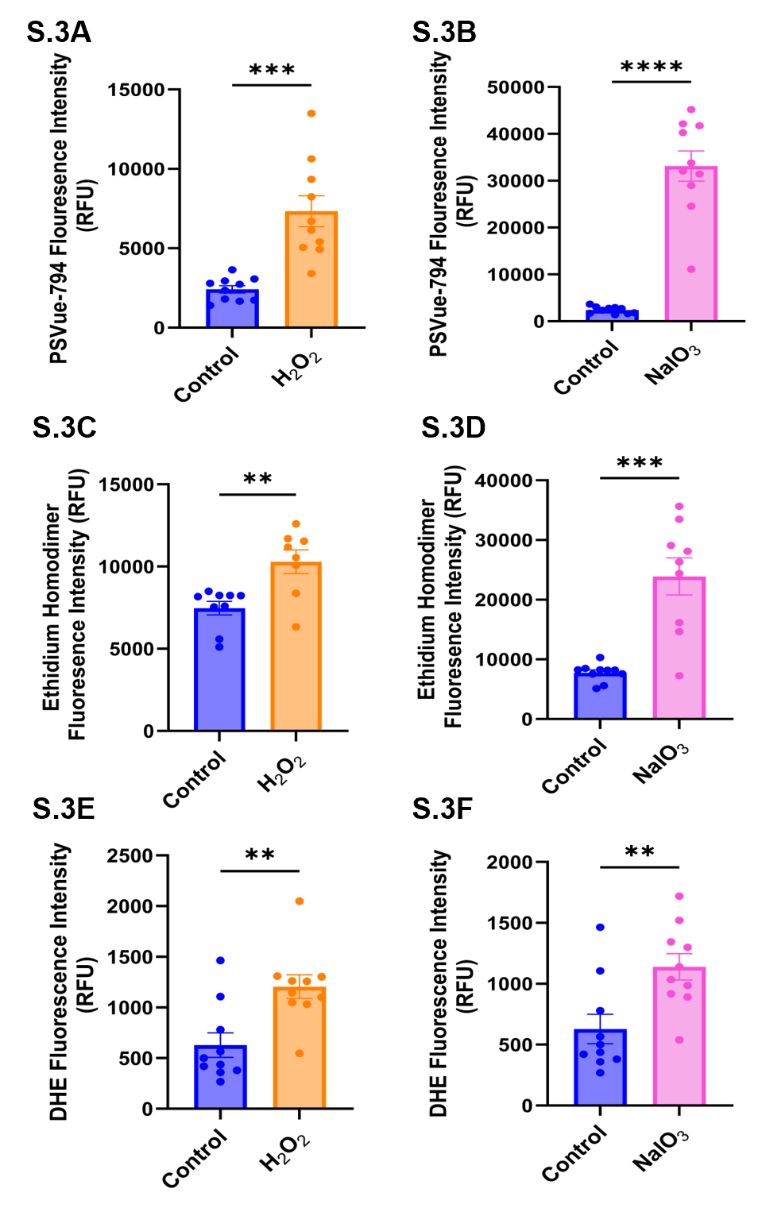


Retinal organoids were treated with hydrogen peroxide (H₂O₂) or sodium iodate (NaIO₃) for 48 h.

1. Live cell death assays using PSVue-794 showed increased cell death in ROs treated with H₂O₂ and NaIO₃ (A and B). Error bars represent Mean±SEM, One-way ANOVA; ***p<0.002; **** p<0.0001. N=8-10 ROs.
2. **Live cell death assays using Ethidium homodimer revealed increased cell death in retinal organoids following treatment with H₂O₂ and NaIO (C and D).** Error bars are represented as Mean±SEM, One-way ANOVA; ** p<0.005; ***p<0.002. N=8-10 ROs.
3. Oxidative stress assays demonstrated elevated ROS levels under H₂O₂ and NaIO₃ treatment (E and F), consistent with responses observed with the CSE-treatment. Data are

shown as mean ± SEM; individual data points represent independent replicates. One-way ANOVA; ** p<0.005; **p<0.005. N=8-10 ROs.

**Figure S4. Proteomic analysis of ROs treated with CSE-750 µg/ml or DMSO for 48 hr.**

**
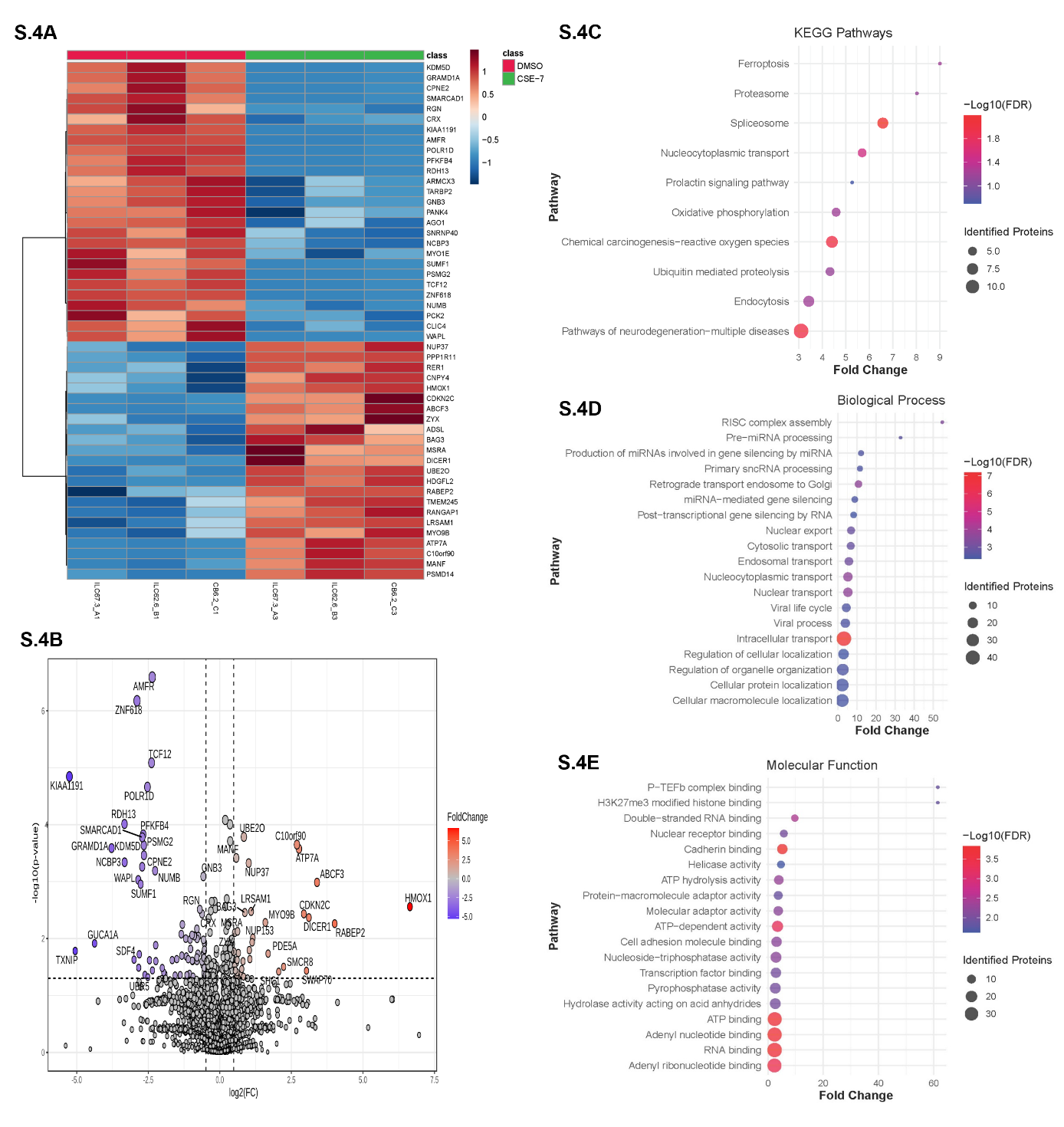
**

LC-MS proteomic analysis of ROs treated with CSE-750 µg/ml show very similar results to those observed in ROs treated with CSE-500 µg/ml, along with several additional DEPs.

- 1. Heatmap and hierarchical clustering representation of the top 50 DEPs across three independent hiPSC cell lines (ILC67.3, ILC62.6, and CB6.2), comparing DMSO and CSE-750 µg/ml treatments. Each column represents an individual sample, and clustering reflects distinct proteomic signatures induced by CSE exposure.
  2. Volcano plot illustrating differential protein expression between CSE (750 µg/mL) and vehicle (DMSO) control-treated ROs. The x-axis represents the log2 fold change (log2FC), and the y-axis represents the statistical significance (−log10 p-value). A total of 80 significant proteins (Fold Change ≥ 1.4, p < 0.05) were plotted. Black dots indicate non-significant changes; blue and red dots indicate significantly downregulated and upregulated proteins, respectively.
  3. KEGG pathway enrichment analysis of DEPs revealed significant enrichment in pathways associated with ferroptosis, metabolic pathways, neurodegeneration, and others in CSE-treated ROs compared to vehicle (DMSO) control-treated ROs.
  4. Gene Ontology (GO) Biological Process analysis identified key functional categories significantly associated with DEPs in CSE-treated ROs, highlighting pathways involved in autophagy, mRNA processing, and cellular processes.
  5. GO Molecular Function analysis revealed enrichment in molecular activities such as antioxidant activity, receptor binding, and protein transport among the DEPs following CSE exposure.

**Table S1. Primary Antibody Information**

| **Primary antibody** | **Company/State/Country** | **Catalog Number** | **Dilution** |
| --- | --- | --- | --- |
| Recoverin | Millipore, Burlington, MA, USA | AB5585 | 1:1000 |
| Human NRL | R&D Systems; NE, Minneapolis, MN, USA. | AF2945 | 1:300 |
| RXR-γ (A-2) | Santa Cruz, CA, USA | Sc-365252 | 1:100 |
| Cleaved Caspase 3 (Asp175) | Cell Signaling Technology; Danvers, MA, USA | 9664 | 1:500 |
| Cleaved Caspase 9 (Asp330) | Cell Signaling Technology; Danvers, MA, USA | 7237 | 1:500 |
| AP-2α | Developmental Studies Hybridoma Bank (DHSB), Iowa, USA | 3B5 | 1:40 |
| OneCut1 (OC1) | Santa Cruz, CA, USA | Sc-13050 | 1:250 |
| PKCα | Proteintech, IL, USA | 2199-1-AP | 1:800 |
| RLBP1 | Proteintech, IL, USA | 15356-1-AP | 1:300 |

**Table S2.** **Secondary Antibody information**

| **Secondary antibody** | **Company** | **Catalog Number** | **Dilution** |
| --- | --- | --- | --- |
| Donkey anti-Rabbit IgG (H+L) Highly Cross-Adsorbed Secondary Antibody, Alexa Fluor™ 594 | Thermo Fisher Scientific; Waltham, MA, USA | A21207 | 1:2000 |
| Donkey anti-Mouse IgG (H+L) Highly Cross-Adsorbed Secondary Antibody, Alexa Fluor™ 594 | Thermo Fisher Scientific; Waltham, MA, USA | A21203 | 1:2000 |
| Donkey anti-Rabbit IgG (H+L) Highly Cross-Adsorbed Secondary Antibody, Alexa Fluor™ 488 | Thermo Fisher Scientific; Waltham, MA, USA | A21206 | 1:2000 |
| Donkey anti-Mouse IgG (H+L) Highly Cross-Adsorbed Secondary Antibody, Alexa Fluor™ 488 | Thermo Fisher Scientific; Waltham, MA, USA | A32766 | 1:2000 |
| Donkey anti-Goat IgG (H+L) Highly Cross-Adsorbed Secondary Antibody, Alexa Fluor™ 647 | Thermo Fisher Scientific; Waltham, MA, USA | A21447 | 1:2000 |
